# Comprehensive Identification of *HD-Zip* Family Genes in *Coix lacryma-jobi* L. and Their Potential Roles in Response to Abiotic Stress

**DOI:** 10.3390/plants14091318

**Published:** 2025-04-26

**Authors:** Yongle Wang, Hongjuan Wang, Xianyong Lu, Chun Yu, Benli Jiang, Jiabao Zhu, Yujiao Wang

**Affiliations:** 1 Institute of Industrial Crops, Anhui Academy of Agricultural Sciences, Hefei 230001, China; 2 School of Life Sciences, Anhui Agricultural University, Hefei 230036, China

**Keywords:** *HD-Zip*, *Coix lacrma-jobi* L., abiotic stress, expression pattern

## Abstract

*HD-Zip* (homeodomain-leucine zipper) transcription factors play a crucial role in plant growth, development, and stress response; however, the *HD-Zip* gene family of *Coix lacryma-jobi* L. has not been identified. In this study, a total of 40 *HD-Zip* gene family members were identified in the genome of Coix. According to phylogenetic analysis, the Coix *HD-Zip* gene was divided into four subfamilies (I–IV), of which the *HD-Zip* I subfamily can be further divided into five branches. Moreover, *HD-Zip* members of the same subfamily usually share similar gene structures and conserved motifs. The transcription factor binding site enrichment analysis showed that there are many motifs for binding with transcription factors such as ERF (Ethylene responsive factor), MYB (v-myb avian myeloblastosis viral oncogene homolog), and ARF (Auxin Response Factor) in the promoter region of the *ClHDZ* genes. The results of qPCR (Quantitative Polymerase Chain Reaction) and expression profile analysis showed that *ClHD-Zip I* genes showed different levels of expression under different stress treatments. Among them, *ClHDZ4* was located in the nucleus, and its expression pattern was significantly upregulated under salt, drought, and high-temperature stress. In addition, ectopic expression of *ClHDZ4* enhanced the growth of yeast strains under drought, salt, or high-temperature treatment. In summary, these results laid a foundation for further research on the resistance function of the Coix *HD-Zip* gene.

## 1. Introduction

**Figure 1:**
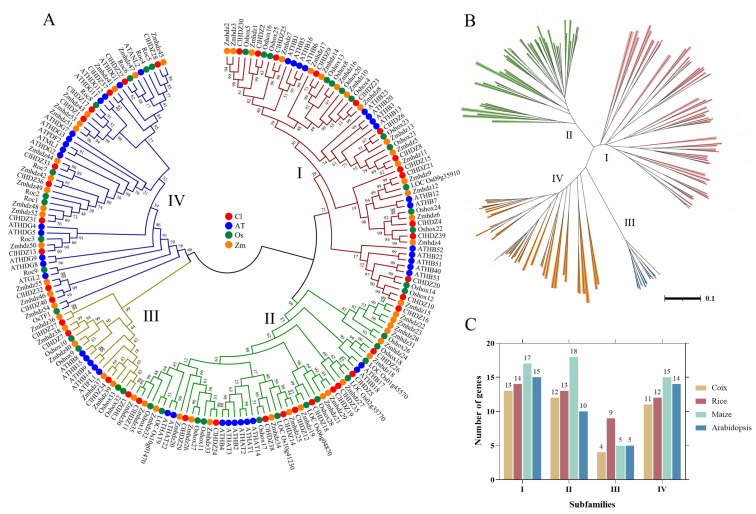
Phylogenetic tree of *HD-Zip* genes from Coix, Arabidopsis, rice, and maize; 40 *ClHDZ* genes, 44 *AtHDZ* genes, 48 *Oshox* genes, and 55 *Zmhdz* genes are clustered into four subgroups (I–IV). *HD-Zip* genes from Coix, Arabidopsis, rice, and maize are denoted by red, blue, green, and *HD-Zip* transcription factors play an important role in shape. The tree was generated with the Clustal X 2.0 software using the neighbor-joining (N-J) method. (**A**) Phylogenetic trees of the *HD-Zip* genes from different species. (**B**) The distribution of the four subfamilies (I–IV) of the *HD-Zip* genes; (**C**) The number of genes in each *HD-Zip* subfamily in different species.

Transcription factors (TFs) are important regulatory factors that control gene expression in plants and play an important regulatory function against various adversity stresses [1]. The homeodomain-leucine zipper (*HD-Zip*) TFs are unique to plants and play an important role in plant adversity signaling and adaptation. The DNA-binding structural domain of *HD-Zip* TFs consists of a homeodomain (HD) and leucine zipper (LZ) [2]. The HD is encoded by HB and generally contains 60 or 61 amino acids for specific interaction with DNA, whereas the LZ is located downstream of HD and is involved in heterodimerization of proteins [3]. Based on variations in protein structure and function, the *HD-Zip* TF family can be divided into four subfamilies, namely *HD-Zip* I, *HD-Zip* II, *HD-Zip* III, and *HD-Zip* IV (Figure 1) [4]. *HD-Zip* I and *HD-Zip* II contain only the basic HD and LZ domains, while *HD-Zip* III and *HD-Zip* IV also have a START domain associated with steroid binding [5,6]. In addition, *HD-Zip* III subfamily proteins contain a specific MEKHLA domain at the C-terminal [7].

It is well known that *HD-Zip* gene family members are involved in the regulation of plant growth and development, such as light response, organ tissue development, shade avoidance response, pigment accumulation, hormone synthesis, and so on [8,9,10]. In maize, *OCLl* from the *HD-Zip* IV subfamily has been identified as a regulator of flowering time [11]. In tomatoes, *SlHB8* from the *HD-Zip* III subfamily has been shown to act as a negative regulator of lignin formation and xylem formation, regulating leaf and stem development [12]. A recent study has identified an *HD-Zip* III TF named *BjPHV* as a negative regulator of non-glandular trichome initiation in *Brassica* L. [13]. Mounting evidence indicates that *HD-Zip* I TFs are widely involved in plant response to abiotic stress [14,15,16]. For instance, overexpression of *MdHB-7* in apples can effectively reduce root damage caused by salt stress and also plays a positive role in ROS regulation. On the contrary, silencing *MdHB-7* can improve the sensitivity of apples to salt stress [17]. Under drought stress, *ZmHB53* overexpressing maize plants showed tolerance during germination, as well as improved seedling drought tolerance [18]. In addition, the *HD-Zip* I TF *ZmHDZ9* enhances drought resistance of maize by regulating the accumulation of abscisic acid and lignin [19]. Further studies showed that lily *LlHB16* promotes thermotolerance, whereas *LlHOX6* interacts with *LlHB16* to limit its transactivation, thereby impairing heat stress responses in lilies [20]. Screening of *HD-Zip* family genes in cucumber has shown that two members of the *HD-Zip* I subfamily, *CsHDZ02* and *CsHDZ33*, are induced to be expressed by high-temperature stress [21].

*Coix lacryma-jobi* L. originated in Southeast Asia and has been cultivated in China for more than six thousand years [22]. Coix is an annual or perennial cash crop, its seeds contain rich nutrients and medicinal components. In addition, Coix has good adaptability to many biological and abiotic stresses, including drought, high temperature, pests, and diseases [23]. At present, the genome sequencing of Coix has been completed [24], which has provided important genome resources for the mining and utilization of genes related to resistance.

However, studies on the function of the *HD-Zip* gene family in Coix are very limited. The aim of this study was to investigate the gene structure, chromosome localization, collinearity, phylogenetic relationship, and TFBS of the Coix *HD-Zip* gene family, and to explore its expression pattern under high temperature, drought, and salt stress. In addition, *ClHDZ4* was selected for further study based on bioinformatics analysis and expression patterns, and the subcellular localization of this protein was determined. Finally, the functional analysis of *ClHDZ4* heterologous expression in yeast was investigated.

## 2. Results

### 2.1. Identification and Characteristic Analysis of HD-Zip Genes in Coix

In this study, 40 *HD-Zip* genes were identified in the Coix genome. According to the Latin abbreviation of Coix and the physical location of the gene, the Coix *HD-Zip* TFs were named *ClHDZ1* to *ClHDZ40* (Table S1). The predicted length of ClHDZ proteins varied from 223 to 883 amino acids (aa) in length, with an average of 481 aa, which is similar to that reported in soybean (462 aa) [25]. Moreover, the isoelectric point (pI) of ClHDZ proteins ranged from 4.58 to 11.44, and their molecular weights ranged from 24 to 96 kDa (Table S1). In addition, the predicted subcellular localization data revealed that most Coix *HD-Zip* proteins were located in the nucleus (Table S1). Table S1 provides information on other *ClHDZ* gene characteristics.

### 2.2. Phylogenetic Trees of HD-Zip Genes in Arabidopsis, Rice, Maize, and Coix

**Figure 2:**
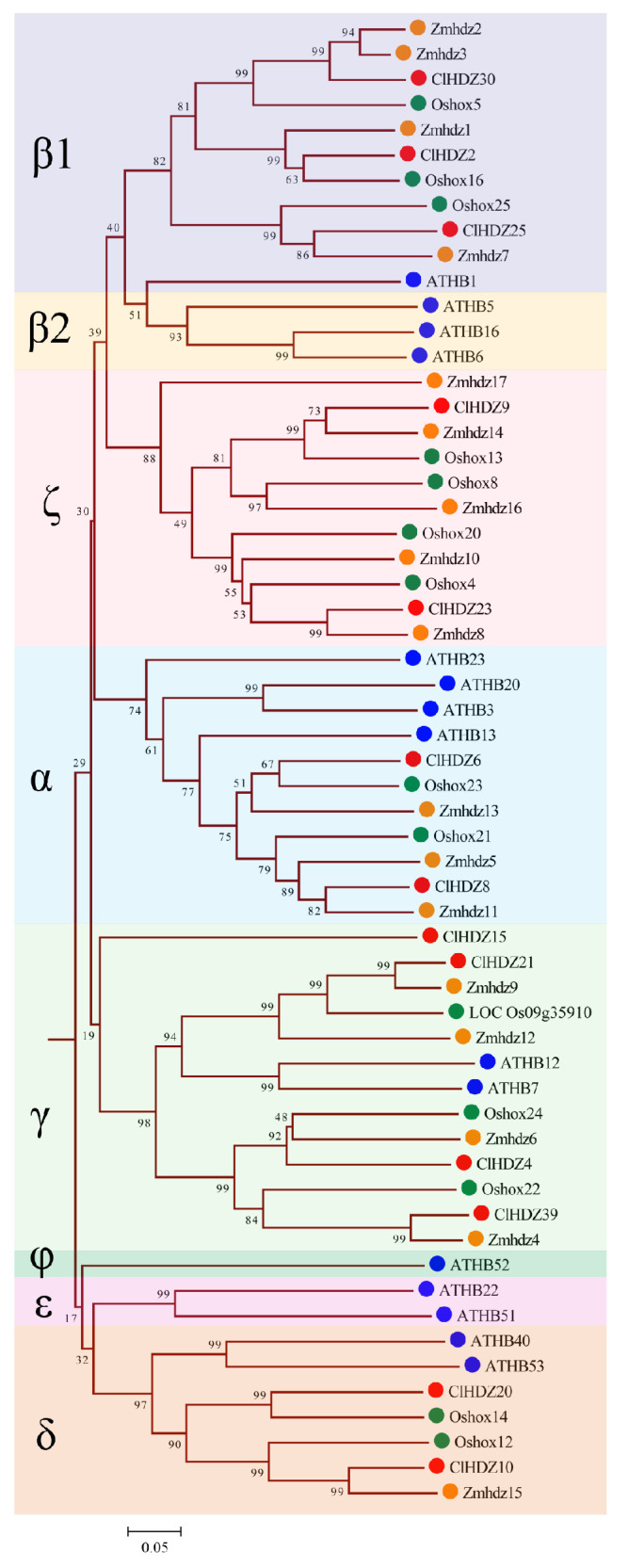
Phylogenetic tree based on *HD-Zip* I protein sequences from Coix, rice, Arabidopsis, and maize. The tree was generated with the MEGA11.0 program using the NJ method. Coix, rice, Arabidopsis, maize, and sorghum *HD-Zip* I proteins are marked with different colored dots. *HD-Zip* I subfamily was further subdivided into eight clades, designated α, β1, β2, γ, δ, ε, ζ, and φ. The red circle represents *ClHDZs*, the blue circle represents ATHBs, the green circle represents Oshoxs, and the orange circle represents Zmhdzs.

For the comparative evolutionary analysis of this gene family in rice, maize, Arabidopsis, and Coix, a phylogenetic tree was constructed using Neighbor-Joining (NJ) methods in MEGA 11.0 (Figure 1). Based on phylogenetic analysis, 187 *HD-Zip* proteins were clearly separated into four subfamilies (*HD-Zip* I to IV), which was in line with earlier discoveries in maize and other species. The results revealed that *HD-Zip* I and *HD-Zip* III subfamilies consisted of the largest and smallest members, respectively, except in maize. Furthermore, 40 Coix *HD-Zip* genes were divided into four subfamilies based on their phylogenetic relationships; subfamily I contained 13 *ClHDZs*, followed by subfamilies II and IV, which had 12 and 11 members, respectively. Furthermore, subfamily I was further subdivided into eight clades, designated α, β1, β2, γ, δ, ε, ζ, and φ, based on the classification of rice, Arabidopsis, and maize (Figure 2). And ζ does not contain the *HD-Zip* gene of Arabidopsis, whereas β2, ε, and φ exclude *HD-Zip* genes from maize, rice, and Coix. Given that Arabidopsis is a dicotyledon, while the other three species are gramineous and monocotyledons.

### 2.3. Chromosomal Distribution and Collinear Analysis

**Figure 3:**
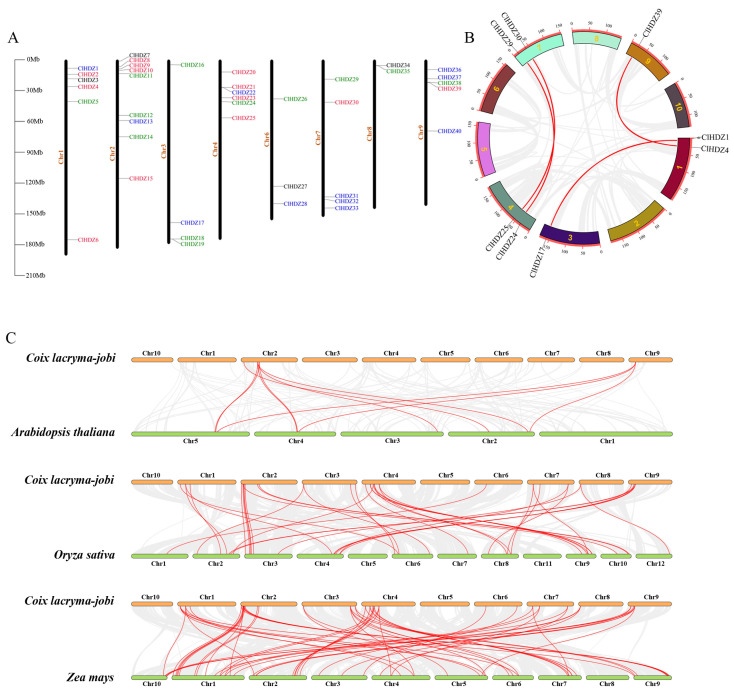
Chromosome distribution and Collinearity analysis. (**A**) Chromosomal location of *HD-Zip* genes in Coix. Different colors represent different subfamilies. (**B**) Collinearity analysis of the *HD-Zip* gene in Coix. (**C**) *HD-Zip* gene collinearity between Coix and other species genomes. The collinear pairs were connected by red lines.

Based on the Coix annotation file, the locations of the 40 Coix *HD-Zips* on the chromosomes were mapped using TBtools v2.082. As shown in Figure 3A, the 40 *ClHDZ* genes were randomly and unevenly distributed on 8 of the 10 chromosomes, except for chromosomes 5 and 10. The result demonstrated that chromosome 2 had the highest quantity of genes, with 9 *ClHDZ* genes, followed by chromosomes 1 and 4, which contained 6 *ClHDZ* genes.

The potential evolutionary mechanism and replication events of the Coix *HD-Zip* gene family were analyzed (Figure 3B), and it was found that there were 4 homologous genes on the Coix chromosomes, namely *ClHDZ1*/*ClHDZ17*, *ClHDZ4*/*ClHDZ39*, *ClHDZ24*/*ClHDZ29*, and *ClHDZ25*/*ClHDZ30*. The results showed that some genes in the Coix *HD-Zip* gene family may have been formed by segmental duplication. Meanwhile, collinearity analysis was performed on the Coix and Arabidopsis genomes, and it was found that there were 10 collinear pairs, and none of the *HD-Zip* III members in Coix were in the collinear regions (Figure 3C). Moreover, a total of 52 collinearity pairs were found between Coix and maize, and the *HD-Zip* genes involved accounted for more than 70% of each genome. Also, 39 collinearity pairs were detected in the Coix and rice (Table S3). These results indicate that the *HDZ* gene families of Coix and maize have a closer homologous evolutionary relationship than that of rice.

### 2.4. Structural Feature Analysis of HD-Zip Genes in Coix

**Figure 4:**
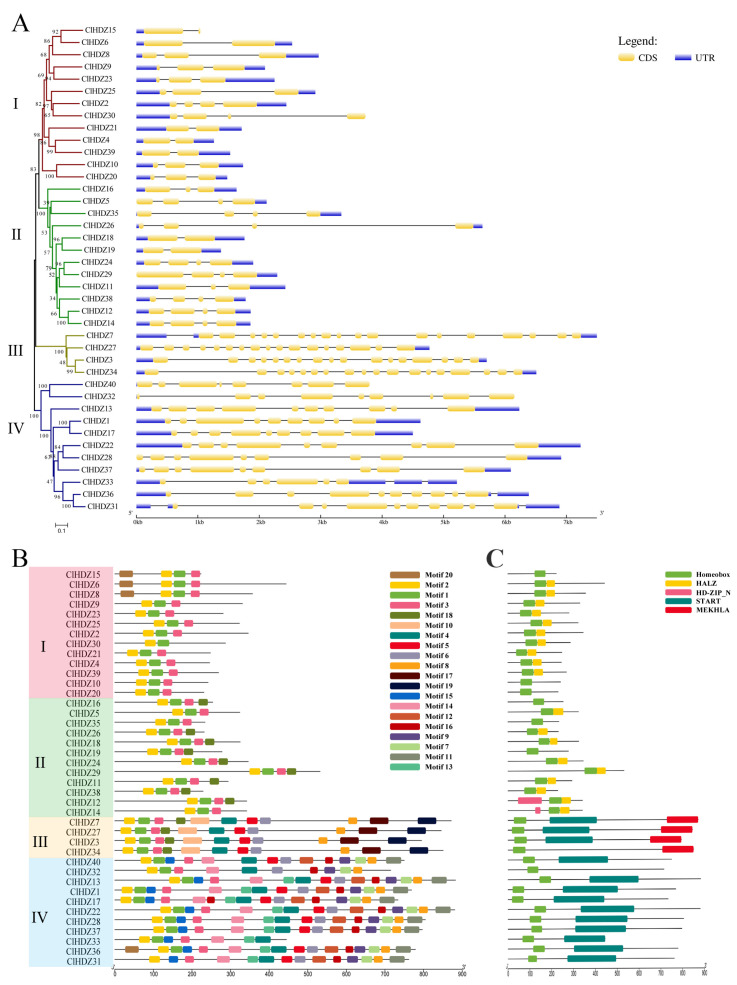
Gene structure and Conserved motifs of *HD-Zip* genes in Coix. (**A**) Exons are indicated by yellow rectangles. Gray lines connecting two exons represent introns. (**B**) Distribution of the 20 conserved motifs in the *ClHDZ* genes following analysis by MEME tool. The different-colored boxes represent different motifs and their position in each protein sequence of *ClHDZ*. (**C**) Distribution of domains in *ClHDZ* genes. Different-colored rectangles indicate the location and distribution of different domains in each *ClHDZ* genes.

Exon/intron structure analysis using the coding sequences of each Coix *HD-Zip* gene was performed in order to gain more information about the structural variety of these genes. In Figure 4A, the exon numbers of the 40 *ClHDZ* genes ranged from 2 to 18. Further comparative analysis showed that genes with close clustering relationships in the evolutionary tree had similar gene structures, and members belonging to the same subfamily shared similar numbers of exons. In the *HD-Zip* I subfamily, most of the members had two or three exons, and the gene structure of the members of this family was simpler than that of the other families. Members of the *HD-Zip* II subfamily shared a similar gene structure with members of the *HD-Zip* I subfamily, and their exon counts are comparable to those of the *HD-Zip* I subfamily. The gene structure of *HD-Zip* III and *HD-Zip* IV subfamily members was relatively complex, and the number of exons in the *HD-Zip* III subfamily was the largest, with 17–18 exons. Members of the *HD-Zip* IV subfamily also had 6–10 exons.

Moreover, we identified the conserved motif of the protein using the Motif Elicitation tool to gain a better understanding of the structural features of the Coix *HD-Zip* genes. As shown in Figure 4B, Motifs 1 and 2 were present in all HDZ proteins, while the ClHDZ31 protein lacked Motif 3. For further functional annotation (Table S4), Motif 1 and Motif 2 were homeobox domains (HD), and Motif 3 was homeobox-associated leucine zipper (HALZ). These three motifs collectively formed conserved motifs of the features of the *HD-Zip* gene family, which was consistent with the structural basis of the classification of the *HD-Zip* family by Ariel et al. (2007) [2]. With the exception of the normal HD domain and LZ domain, the results demonstrated that the *HD-Zip* I and *HD-Zip* II subfamily members shared similar structures and comprised only a small number of simple motifs. *HD-Zip* III and *HD-Zip* IV subfamily members have more motifs and more complex domains. The START domain, which was unique to *HD-Zip* III and *HD-Zip* IV subfamily members, was made up of Motifs 4, 5, 6, 10, 13, and 14. Only the *HD-Zip* III subfamily had the MEKHLA domain, which was formed by motif 19. The significant differences in gene structure and domain across the four subfamilies may be related to their different functions in plant growth and development and response to environmental stress.

### 2.5. Prediction and Enrichment Analysis of Transcription Factor Binding Sites in Coix HD-Zip

The transcription factor binding sites (TFBS) of *ClHDZ* were analyzed using the JASPAR database and MEME FIMO (Table S5). A total of 702 TFBS were identified in the promoter sequence of the *ClHDZ*. Enrichment analysis of these TFBS (Tables S1 and S6) revealed that 501 motifs in the promoter sequence had high statistical significance (*p*-value < 0.05), belonging to 32 transcription factor families. Among them, the number of ERF transcription factor family is the largest, with 60. It is noteworthy that BAD1 (MA2408.1), which belongs to the TCP transcription factor family, had the highest odds_ratio (189.86). This motif was significantly enriched in the promoter sequence of *ClHDZ*.

The analysis of the significantly enriched motifs in the *ClHDZ* promoter sequence (Table S7 and Figure S1) revealed that Zm00001d020267 (MA1817.2), which belonged to the ERF transcription factor family, was the most abundant in the *ClHDZ* promoter sequence. Notably, some transcription factors (such as ERF, MYB, and ARF) were significantly enriched in the promoter sequence of the *ClHDZ*, suggesting that these TFs might have played important roles in the regulatory network of *ClHDZ*.

### 2.6. The Expression Files of Coix HD-Zip Genes in Response to Drought and Heat Stress

**Figure 5:**
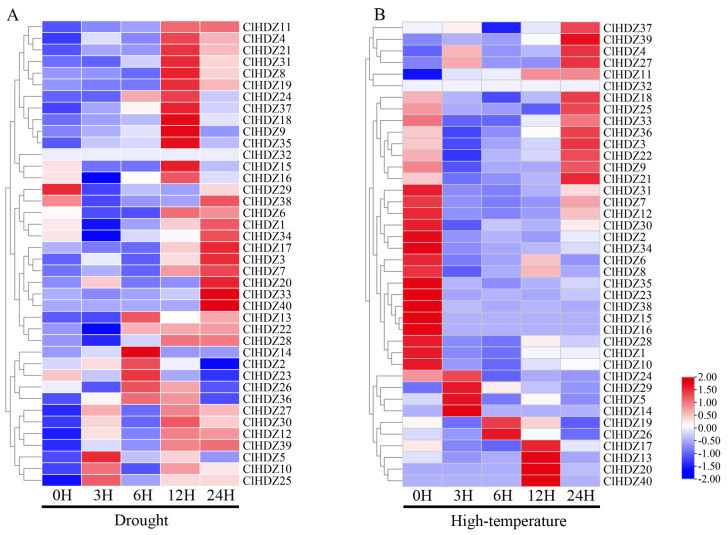
Heat map of *HD-Zip* gene expression pattern in Coix. (**A**) The expression levels of *ClHDZ* under drought stress. (**B**) The expression levels of *ClHDZ* at different periods of high-temperature stress.

To investigate the potential roles of Coix *HD-Zip* genes in drought and high temperature, we collected two transcriptome datasets for all 40 *ClHDZ* genes from the NCBI database and generated a heat map. During drought stress (Figure 5A), the expression profiles of 11 *ClHDZ* genes were significantly up-regulated at 12 h; whereas 9 *ClHDZ* genes were significantly up-regulated at 24 h of treatment; additionally, one gene (*ClHDZ32*) was found to have no expression information. As for high-temperature stress (Figure 5B), the expression of nine *ClHDZ* genes was apparently down-regulated at any time. The expression of most *ClHDZs* was increased by high-temperature induction, and there were three main expression modes: six *ClHDZs* were rapidly induced and up-regulated at 1 h or 3 h; *ClHDZ17*, *ClHDZ13*, *ClHDZ20*, and *ClHDZ40* were up-regulated at 12 h; and the expression level of 12 *ClHDZs* was significantly up-regulated at 24 h.

### 2.7. Expression Pattern of the HD-Zip I Genes in Coix Under Salt Stress

**Figure 6:**
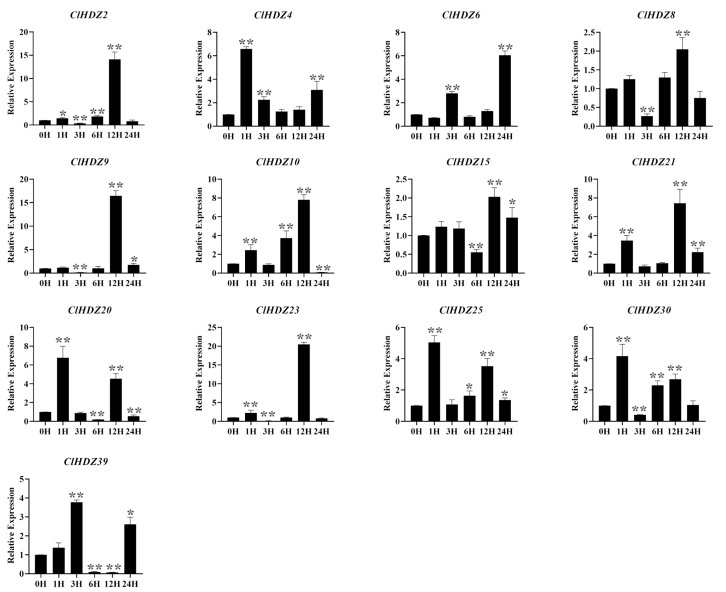
Expression analysis of 13 *HD-Zip I* genes in Coix following salt treatments by qRT-PCR. *Y*-axis and *X*-axis indicate relative expression levels and the time courses of stress treatments, respectively. Mean values and standard deviations (SDs) were obtained from three biological and three technical replicates. The error bars indicate standard deviation. Significance levels: * *p* < 0.05; ** *p* < 0.01.

According to reports, *HD-Zip I* genes played an important role in responses and tolerance to various abiotic stresses, especially drought and salt stress. The expression pattern of *HD-Zip I* genes in NaCl-treated Coix was detected by qRT-PCR (Figure 6). As shown in the figure, the expression levels of four genes rapidly responded and peaked at 1 h, among which the expression levels of *ClHDZ4* and *ClHDZ20* reached a maximum value of more than 6-fold. Furthermore, *ClHDZ25*/*ClHDZ30* exhibited a similar expression pattern after NaCl treatment. For example, the expression of *ClHDZ25* was up-regulated and reached a maximum at 12 h, but then decreased and gradually up-regulated threefold at 12 h. Additionally, the expression level of 7 *ClHDZ* genes exhibited a rapid and strong up-regulation at 12 h.

### 2.8. Subcellular Localization of ClHDZ4 and Its Role in Enhancing Yeast Stress Tolerance Through Ectopic Expression

**Figure 7:**
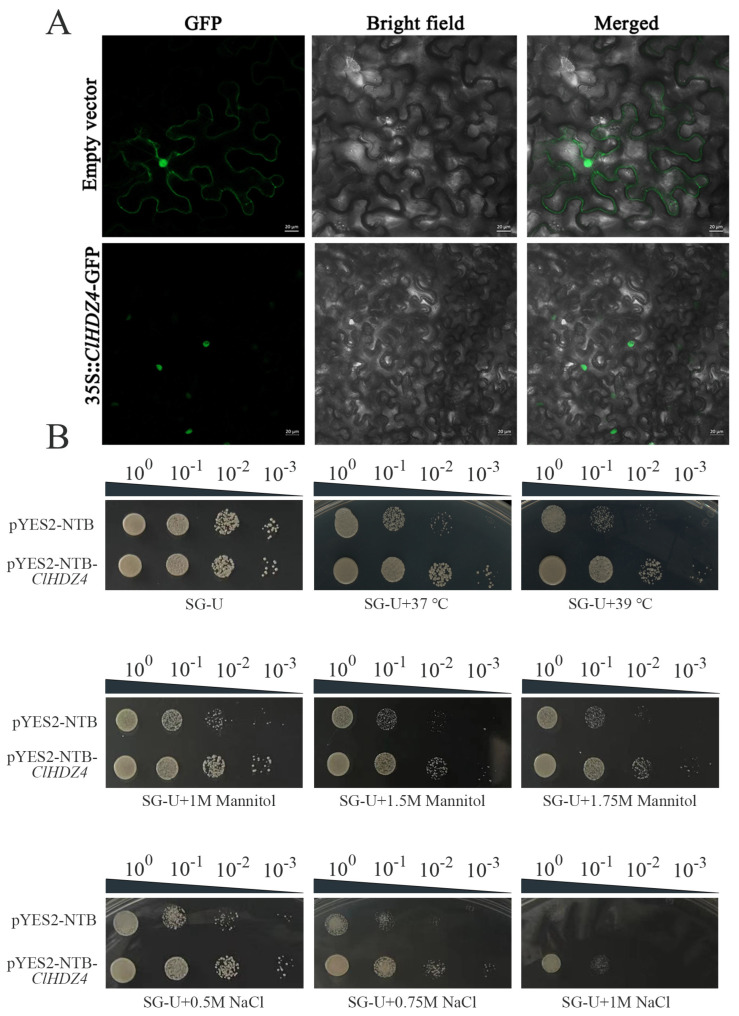
Subcellular localization and detection of drought, NaCl, and high-temperature tolerance of the yeasts transformed with *ClHDZ4*. (**A**) Subcellular localization of *ClHDZ4* in tobacco leaves. (**B**) Growth of the INVSc1 yeast strain transformed with *ClHDZ4*.

The subcellular localization of *ClHDZ4* was identified by constructing a recombinant plasmid p1305-CaMV35S-*ClHDZ4*-GFP and expressing it in the lower epidermis of tobacco leaves. As shown in Figure 7A, fusion fluorescence signals on the nucleus were observed, whereas the control group (p1305-CaMV35S-GFP) exhibited in both the cytoplasm and the nucleus.

In order to investigate whether *ClHDZ4* responds to drought, salt, and high-temperature stress, the effects of *ClHDZ4* on yeast growth and stress resistance were analyzed in yeast containing the pYES2::*ClHDZ4* vector (Figure 7B). According to the comparison with a control yeast strain, we determined whether *ClHDZ4* can enhance the drought, salt, and high-temperature resistance of the yeast strain. Under normal conditions, there was no significant difference in strain growth size between yeast overexpressing *ClHDZ4* and empty vector. However, with the increase in PEG and NaCl concentrations in the medium, the growth of control yeast cells was significantly inhibited. Under 1M NaCl treatment, the yeast strain containing *ClHDZ4* grew better than the control, and the control yeast did not grow at 10-1-fold dilution. Similar results were observed under drought treatment; the growth and survival rate of the transgenic yeast cells harboring the *ClHDZ4* gene was improved. With regard to heat tolerance function, the growth of yeast cells harboring the *ClHDZ4* gene was improved under 37 °C treatment compared to the control. When the temperature was increased to 39 °C, the growth of continuously diluted control yeast cells on SD/-Ura medium was significantly inhibited. These results indicate that the *ClHDZ4* gene enhanced the resistance of yeast cells to drought, NaCl, and high temperature.

## 3. Discussion

Plants are regulated by a variety of TFs in the process of growth and development, and *HD-Zip* TFs are a plant-specific gene family whose functions have been widely reported in plants such as Arabidopsis [26], rice (*Oryza sativa* L.) [27] and maize (*Zea mays* L.) [28]. Hence, 40 Coix *HD-Zip* genes were genome-wide identified and characterized in this study.

Coix *HD-Zip* genes could be categorized into four subfamilies according to their structure, and the number of members of each subfamily shows variability in different species. For example, the number of members corresponding to the four subfamilies *HD-Zip* I to IV in Coix was 13, 12, 4, and 11, respectively, while the number of members of the four subfamilies in maize was 17, 18, 5, and 15, respectively (Figure 1). It is well known that *HD-Zip I* subfamily genes can be further divided into 8 branches (α, β1, β2, γ, ε, δ, ζ, and φ) [29]. In the present study, phylogenetic analysis showed that the 13 *HD-Zip* I proteins of Coix were divided into five clades without the φ, ε, and β2 clades, indicating that Coix had lost members of these three clades during evolution. Similarly, in monocotyledons such as rice [27], maize [28], and perennial ryegrass (*Lolium perenne* L.) [30], it had been reported that φ, ε, and β2 evolutionary branches of *HD-Zip* I proteins were absent. Comparative evolutionary analysis showed that the phylogenetic relationship of the *HD-Zip* genes was highly consistent with the relative relationship between these species.

Analysis of gene structure and conserved motifs in Coix showed that members of the same subfamily shared similar motif distribution and exon number, which was consistent with observations made with respect to other species. In Figure 4, *ClHDZs* of subfamily III had the largest number of exons and domains. Moreover, the *HD-Zip* III subfamilies identified in cucumber, watermelon, and kiwifruit [21,31,32] contained the lowest number of genes, but they played important roles in plant growth and development, such as the formation of apical meristematic tissue and vascular system [2]. The similarity of structures indicates that they may perform similar functions. The *ClHDZ4* gene screened in this study had a similar structure and the same number of exons as *Oshox22/24* in rice [27] and *Zmhdz4/6* in maize [28], and all five genes belonged to the γ branch of the *HD-Zip* I subfamily, which was hypothesized to have similar functions.

Plants adapt to environmental stresses by inducing the expression of stress-related genes, such as drought, and the expression of *HD-Zip I* genes in many species responds to drought treatment and plays an important role in drought stress [14,33]. Analysis of transcription factor binding sites in the promoter region of the ClHDZ genes showed that the promoter region contains multiple motifs that bind to ERF, MYB, and ARF transcription factors. According to the literature, ERF, MYB, and ARF are widely involved in regulating plant responses to abiotic stresses [34,35,36]. The TFBS enrichment analysis results show that the BAD1 (MA2408.1) motif, which belongs to the TCP transcription factor family, has the highest odds ratio in the ClHDZ promoter. This suggests that the TCP transcription factor BAD1 was likely to be an important regulator of ClHDZ. It has been reported that the TCP transcription factor family plays an important role in the plant response to drought stress [37,38]. Therefore, the expression level of the ClHDZ genes under drought stress was investigated. The results showed that members of the *HD-Zip* I subfamily in Coix responded positively to drought stress, which was the same as that in Arabidopsis, wheat, and apple [17,39,40]. It was found that *AtHB12*, a member of the γ branch of *HD-Zip* I subfamily, was induced by drought stress, and overexpression of *AtHB12* enhanced the tolerance of transgenic Arabidopsis to drought stress [41]. Similarly, *Md HB-7* and *Md HB7-like*, which were members of the γ branch of the *HD-Zip* I subfamily, enhanced the drought adaptation and water-use efficiency of transgenic apple by regulating the stomatal density [17,42]. Given this, members of this branch might play an important role in drought stress response. The accumulated studies revealed that *HD-Zip* I TFs were also involved in the regulation of salt and high-temperature stresses [43]. For example, *CaHDZ15* (*HD-Zip I* gene) in pepper [44] directly targeted and activated heat shock factor *A6a* (*HSFA6a*), which further activated *CaHSFA2*, and its overexpression significantly improved the tolerance of transgenic tobacco to high-temperature stress. In *Sophora alopecuroides*, overexpression of *SaHDZ22* [45] increased Arabidopsis tolerance to salt stress. In this study, the expression level of *HD-Zip I* genes was also induced under high-temperature stress and salt stress. Among them, *ClHDZ4* has a collinear relationship with *Zmhdz4* and *Zmhdz6*. Moreover, overexpression of *Zmhdz4* has been reported to enhance drought resistance of transgenic maize plants [46]. In addition, *AtHB7*, *AtHB12*, and *ClHDZ4* belong to the same clade, and the expression levels of *ATHB7* and *ATHB12* were also significantly up-regulated under NaCl and drought stress [39]. The expression level of *ClHDZ4* was up-regulated under drought, salt, and high-temperature stress, and its overexpression could enhance the stress resistance of yeast strains.

## 4. Materials and Methods

### 4.1. Retrieving and Identifying HD-Zip Family Genes in Coix

The genome annotations of the Coix were fetched from Coge (https://genomevolution.org/coge/, accessed on 21 February 2025). The accession numbers for the HD-domain profile (PF00046) and LZ domain profile (PF02183) were downloaded from InterPro (https://www.ebi.ac.uk/interpro/entry/pfam/, accessed on 21 February 2025). Subsequently, their Hidden Markov Model (HMM) profiles were used to search the Coix protein database via the HMMER program with an E-value less than 1 × 10^−5^ as the threshold [47]. The candidate *HD-Zip* protein sequences underwent verification through the SMART (http://smart.embl.de, accessed on 21 February 2025) and CDD database on the NCBI website (https://www.ncbi.nlm.nih.gov/cdd, accessed on 21 February 2025). Protein properties were obtained via EXPASY (https://web.expasy.org/protparam/, accessed on 21 February 2025), including theoretical isoelectric points (pI) and molecular weights (Mw). The online Cell-PLoc server and CELLO v.2.5 (Plant-PLoc, http://www.csbio.sjtu.edu.cn/bioinf/plant/ (accessed on 21 February 2025) and http://cello.life.nctu.edu.tw/ (accessed on 21 February 2025)) were used to predict the subcellular localization of *HD-Zip* proteins in Coix [48].

### 4.2. Phylogenetic Analysis, Chromosome Localization, and Collinear Analysis

The multiple alignment of 40 Coix *HD-Zip* proteins, 44 Arabidopsis *HD-Zip* proteins, and 48 rice *HD-Zip* proteins was performed using the ClustalW program in MEGA7.0 with default parameters. Next, MEGA11.0 was used to create a phylogenetic tree using the neighbor-joining method (NJ) with 1000 bootstrap replicates and default parameters [49].

The GFF annotation file from the Coge database provided the chromosomal location data for the Coix *HD-Zip* genes. The Gene Location Visualize feature in the Tbtools software was used to visualize the physical maps of the Coix *HD-Zip* genes. To conduct inter-species collinearity analysis, genome sequences and annotation files for Arabidopsis, maize, and rice were obtained from the Phytozome v13 website. The collinearity relationships between Coix and three other species were analyzed and visualized using TBtool [50].

### 4.3. Gene Structure, Conserved Motifs, Transcription Factor Binding Site (TFBS) Analysis, and Enrichment Analysis

Based on the exon and intron positions of *HD-Zip* genes in the GFF annotation file of the Coix genome, the intron-exon structure was analyzed and mapped using TBtools software. The Multiple Expectation Maximization for Motif Elucidation (MEME) tool in TBtools v2.082 software was used to predict and analyze the conserved protein motif of Coix seed *HD-Zip* protein, with the maximum number of motifs set to 20, and other parameters set to default.

The promoter sequences of all protein-coding genes of *Arabidopsis thaliana* were downloaded from the EPD database (https://epd.expasy.org/epd/, accessed on 22 February 2025) as the control set, while the 2000 bp sequence upstream of the ClHDZ genes promoter was used as the test set [51].

The TFBS in both the test and control sets were predicted using the JASPAR database (https://jaspar.elixir.no/, accessed on 22 February 2025) and the FIMO tool in the MEME Suite (https://meme-suite.org/meme/tools/fimo, accessed on 22 February 2025), with a Match *p*-value threshold of <1 × 10^−4^ [52]. Subsequently, Fisher’s exact test was applied to the results for enrichment analysis, retaining only the motifs with a *p*-value < 0.05.

### 4.4. Expression Profiles of HD-Zip Genes in Coix

The expression profile of the *HD-Zip* gene in Coix under high-temperature and drought stress was analyzed using Coixtranscriptome data (NCBI BioProject: PRJNA812268), the mean TPM values of three replicates were calculated, and the TBtools software was used to draw a heatmap of gene expression.

### 4.5. Plant Material Acquisition, RNA Extraction, and qRT-PCR

The Wanyi 2 variety of Coix was cultured in the growth chambers at a temperature of 26 °C with a light/dark cycle of 16/8 h. Before the salt treatment, seedlings were pre-cultivated in 1/2 Hoagland nutrient solution for one month. Subsequently, it was treated with 200 mM NaCl, and samples were taken at day 0, 1, 3, 6, 12, and 24. Additionally, the samples were kept at −80 °C and frozen with liquid nitrogen. The RNA of the samples was extracted using the Aidlab plant RNA kit (Aidlab Biotech, Beijing, China), and the first-strand of cDNA was synthesized using the Prime ScriptTMRT reagent Kit (TaKaRa, Dalian, China). Real-time PCR was performed by CFX96TM Real-Time System and using TB Green Premix Ex Taq II qRT-PCR with a sample volume of 10 μL. The relative expression levels of each gene were determined using the standard 2^−ΔΔCT^ method [53]. *VQ30* was utilized as a reference gene, and specific primers for Coix *HD-Zip* genes were designed by Primer Premier 5.0 software.

### 4.6. Subcellular Localization Analysis of ClHDZ4

The coding sequence of *ClHDZ4* (without the stop codon) was amplified with the primer pairs (Supplementary Table S2) and then cloned into a pCambia1305-35S::GFP vector fused with the N-terminus of the green fluorescent protein (GFP) gene. The GFP-only control vector and *ClHDZ4*-GFP vector were transformed into the Agrobacterium strain GV3101 and injected into the tobacco leaves for transient expression. Tobacco was grown in a greenhouse with a 16/8 h light and dark photoperiod at 24 °C for 4 weeks. After 48 h, the GFP fluorescence was detected by a laser scanning confocal microscope (Leica TCS SP8, Wetzlar, Germany).

### 4.7. The Overexpression of ClHDZ4 in Yeast

The coding sequence of *ClHDZ4* was cloned into the pYES2/NTB (pYES2) vector and then transformed into the yeast strain INVSc1. Yeast monoclonal colonies were selected and confirmed through PCR analysis. Transformed yeast cells were incubated in SD-Ura liquid medium at 29 °C overnight until the optical density (OD)600 reached 1.2, then transformed yeast cells were shake-cultured at 250 rpm, at 29 °C, for 10 h in liquid SG-U medium. The yeast (containing pYES2 empty liquid or pYES2::*ClHDZ4* recombinant vector) was diluted to 1:10, 100, 1000, and 10,000. Then, 4 μL of the original yeast liquid and the diluted yeast liquid were successively dropped on the SG-U solid medium. For drought and salt treatments, we added different concentrations of PEG or NaCl to the SG-U medium, while for high-temperature treatments, the SG-U solid medium was incubated for 7 days at 37 °C and 40 °C.

## 5. Conclusions

In this study, 40 *HD-Zip* genes of Coix were identified, which were classified into four subfamilies according to phylogenetic analysis and conserved domains. The Coix *HD-Zip* genes were randomly and unevenly distributed on 8 of the 10 chromosomes, all of which were predicted to be located in the nucleus. Moreover, *ClHDZ* genes showed different expression patterns under different stress treatments (drought, salt, and high temperature). In addition, ectopic expression of *ClHDZ4* enhanced the growth of the yeast strain under drought, salt, or high-temperature treatment. Overall, the results of this study are helpful to further elucidate the regulatory mechanism of the *HD-Zip* gene in response to abiotic stress and provide reference information for genetic breeding.

## Data Availability

All data in this study can be found in the manuscript or the Supplementary Materials.

## Supplementary Materials

The following supporting information can be downloaded at: https://www.mdpi.com/article/10.3390/plants14091318/s1. Table S1. Details of the identified Coix *HD-Zip* genes. Table S2. List of primer sequences used for experiments in this study; Table S3. Collinearity analysis of *HD-Zip* gene between Coix and other species; Table S4. Detailed information for the 30 motifs in the *HD-Zip* proteins of Coix; Table S5. Prediction of Transcription Factor Binding Sites in *ClHDZ*. Table S6. Enrichment analysis of transcription factor binding sites of *ClHDZ*. Table S7. Number of binding sites for each transcription factor in *ClHDZ*. Figure S1. Heat map analysis of the number of binding sites for each transcription factor in *ClHDZ*.
