# Supplementary material for: Comprehensive Identification of HD-Zip Family Genes in Coix lacryma-jobi L. and Their Potential Roles in Response to Abiotic Stress
Source: Plants (Basel). 2025 Apr 26;14(9):1318. doi: 10.3390/plants14091318 (PMC12073142; doi:10.3390/plants14091318)

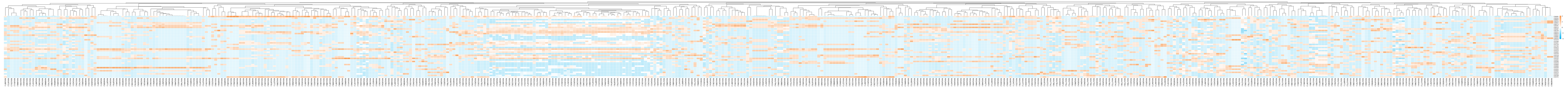

Supplement: Supplementary file 1 [file Figure S1.pdf › Figure S1.pdf]
